# Information Needs of State-Level Asthma Programs: Recommendations to Increase Accessibility

**DOI:** 10.3390/ijerph21121670

**Published:** 2024-12-14

**Authors:** Mary Fox, Ann Joseph, Neha Parimi, Haley Huh

**Affiliations:** 1 Department of Health Policy and Management, Johns Hopkins Bloomberg School of Public Health, Baltimore, MD 21205, USA; 2 Baltimore City Health Department, Baltimore, MD 21202, USA; niajoseph@gmail.com; 3 Division of Pediatric Endocrinology, Johns Hopkins School of Medicine, Baltimore, MD 21287, USA; nparimi1@jhmi.edu; 4 College of Arts and Sciences, Emory University, Atlanta, GA 30322, USA; haleyhuh@gmail.com

**Keywords:** accessibility, asthma, state government program, health education

## Abstract

Asthma is the most common pediatric chronic disease, affecting about 5 million U.S. children. Controlling asthma requires intervention at the individual, household, and community levels. State asthma programs offer activities to support asthma control. We profiled U.S. state asthma programs through a website survey (24 CDC-funded, 10 non-CDC-funded) and then interviewed program managers about their information needs to identify ways to support their work. While CDC-funded and non-CDC-funded programs report similar goals and activities, their levels of engagement differed. Most programs rely on federal agencies or national associations for education materials. Concerns about materials were the lack of accessibility due to readability (reading level above sixth grade) and the lack of translations for culturally and linguistically diverse populations. In addition to priority needs around accessibility of materials, programs requested research to enhance their work and to support asthma management for young adults and incarcerated individuals.

## 1. Introduction

Asthma is a long-term respiratory illness that imposes health and economic difficulties on millions of people globally [1,2,3,4]. In the United States (U.S.), it is the most common pediatric chronic disease, affecting 4.6 million children [5]. Certain groups, such as African Americans, Native Americans, Alaska Natives, and those living in poverty, are more likely to develop asthma [5]. Each year millions of individuals experience asthma attacks and about one million people need emergency care and/or hospitalization [5]. Asthma has a significant economic cost, generating over USD 80 billion in healthcare expenses, and causes productivity loss and premature deaths each year in the U.S. [6]. Asthma is a climate-sensitive disease; higher temperatures, extended pollen seasons, and other climate-related stressors are expected to contribute to an additional disease burden [7,8].

The Global Initiative for Asthma (GINA) develops evidence-based guidance for a comprehensive approach to asthma control and prevention with an emphasis on treatment and patient education for self-management [9]. National and sub-national programs strive to use and disseminate evidence-based guidance with attention to home and ambient environments, which may present asthma triggers, and patient supports found in the community (e.g., schools) and healthcare systems [3,10]. In the U.S., the Center for Disease Control and Prevention’s (CDC) National Asthma Control Program has been in operation for 25 years and has supported sub-national (state, local, tribal, and territorial) asthma programs [10,11]. U.S. statistics show success in asthma control and prevention across several metrics, e.g., declines in asthma prevalence, emergency department visits and mortality in all age groups suggesting that these programs are reducing asthma’s public health burdens [12]. As shown below, all of these sub-national programs use communication to engage one or more audiences; communication is one essential component of successful public health programs [13]. Yet, disparities persist in the U.S. and asthma prevalence in adults has increased [5,12].

The Bridging Research, Lung Health, and the Environment (BREATHE) Center at Johns Hopkins University is one of six Collaborative Centers in Children’s Environmental Health Research and Translation (CEHRT) established to promote interdisciplinary research on the impact of environmental factors on children’s health and to translate scientific findings into policies and interventions [14]. This work was conducted as part of the BREATHE Center’s translation effort. Focused on state programs in the U.S., this project will help the collaborative Centers and the public health community plan asthma-related activities and communication strategies.

State and local health departments have been called the “backbone” of the U.S. public health system [15]. This work explores the state health department role(s) in asthma prevention and control. There were two objectives:

1. To document the goals and activities of state government asthma programs; and
2. To interview asthma program managers about their information gathering, use and needs.

## 2. Materials and Methods

A website review was conducted to gather data on objective 1, followed by interviews. This work was evaluated by the Bloomberg School of Public Health IRB and determined to be “Not Human Subjects Research” (IRB#24225), as this work relies on key informant interviews with asthma program officials about asthma programs.

The sample of programs included in the study was identified in two ways. The CDC currently funds 24 states or territories, and these programs are identified on the CDC website [11]. For the remaining states, the health department websites were searched to find asthma programs. Ten programs currently not receiving CDC funding were found (some of these state programs had CDC funding in the past).

To organize the website survey, the asthma programs were divided into two categories: CDC-funded and non-CDC-funded. Health department websites were then searched for the current asthma program (or program plan), and each website or plan was reviewed using a pre-determined set of questions (Appendix A) to document program work. Program contacts were identified during the website review. Website review information collected was recorded in a spreadsheet. The research team met weekly to review progress and ensure consistent practices. When the website reviews were complete, the study team began contacting program contacts to schedule interviews. Each program contact was emailed. In cases of no response, two more email reminders were sent at weekly intervals. If there was no response after repeated emails, telephone contact was attempted. If there was no response after three calls (conducted at weekly intervals), programs were excluded. During interviews, the study team took notes to record responses. The website survey was conducted from October to December 2022 and interviews took place from March to June 2023. See Appendix B for the interview discussion guide.

## 3. Results

### 3.1. Website Survey

#### 3.1.1. Website Survey Overview

Websites were reviewed for all 24 CDC-funded state programs. Ten non-CDC funded asthma program websites were found and reviewed. The program website URLs are provided in Supplemental Materials (Table S1).

#### 3.1.2. Website Survey: Program Goals and Activities

The websites documented a variety of program goals and activities for both CDC-funded and non-CDC-funded programs, see Table 1. The program elements included in Table 1 were categorized and compiled as described on the websites as they were reviewed. The original data are provided in Supplemental Materials (Spreadsheet S1).

A higher percentage of CDC-funded program websites reported goals of reducing morbidity and mortality, improving quality of life for asthma patients, and reducing disparities. A higher percentage of non-CDC-funded program websites reported goals of improving asthma self-management, reducing school absenteeism, improving access and affordability of care. The proportion of programs that had the goal to reduce ED visits and hospitalizations was similar (+/− 5%).

In terms of activities, a higher percentage of CDC-funded program websites reported providing education and resources, performing in-home visits, having a surveillance system, performing community, school, or provider outreach, and developing policies and guidelines. A higher percentage of non-CDC-funded program websites reported activities related to reducing environmental triggers and developing collaborations. The extent of program engagement was similar (+/− 5%) for improving indoor air/reducing tobacco use.

### 3.2. Interviews

#### 3.2.1. Interview Overview

Thirteen (54%) of the 24 CDC-funded programs were interviewed; the others were excluded due to non-response. One (10%) non-CDC-funded program was interviewed. One (10%) non-CDC-funded health department responded by email that the asthma program had been discontinued due to a lack of funding. The other eight (80%) non-CDC-funded programs did not respond. Interviewees did not identify major gaps in website information. The interview notes are provided in Supplemental Materials (Document S1).

#### 3.2.2. Interview Results: Resources and Information

Most programs gathered resources and information from federal agencies, such as the CDC, the National Institutes of Health (NIH), and the Environmental Protection Agency (EPA). Information was typically obtained from newsletters, reports, and scientific publications. Program staff mentioned that reliance on CDC materials helps ensure that program work aligns with best practices and recommendations. Most programs also reported gathering information from asthma and allergy organizations or through attendance at their meetings. The organizations that were reported included the American Lung Association, the Allergy and Asthma Network, the American Academy of Allergy, Asthma & Immunology, the American Association for Respiratory Care, and the Asthma and Allergy Foundation of America. One program reported using materials from the Green and Healthy Homes Initiative. One program reported using their own surveillance data to guide interventions and gather information about the social determinants of health through their home visiting program (facilitating referral to other services if indicated). Another program reported relying on the Behavioral Risk Factor Surveillance System and health insurance for data on asthma-related morbidity and mortality. One program mentioned gathering information through meetings with other asthma programs.

#### 3.2.3. Interview Results: Concerns and Needs

Many programs expressed concern that the materials they use are outdated and potentially inaccurate. Most programs asked for education resources at the sixth-grade reading level or below in the form of PDF documents and shareable educational videos. Videos for children were a common request. These resources should be culturally sensitive and available in multiple languages appropriate to the local context, including tribes and other prevalent ethnic populations. Several programs requested information and materials related to: air quality; wildfire smoke; and advice on air purifiers (effectiveness, identifying a good quality air purifier, whether an air purifier purchase can be covered by insurance for people with chronic conditions). Also requested were materials to assist physicians with educating patients on air quality, such as how to use air quality alert apps.

Requests made by individual programs for information:

- On the pathophysiology of asthma and on asthma medications;
- On adult asthma management, as well as how to differentiate between asthma and other chronic lung conditions;
- On the transition period between pediatric and adult asthma care;
- On asthma management tailored for incarcerated individuals, especially as they transition back to their communities;
- On home or property maintenance to reduce asthma triggers for homeowners, landlords, and property managers. There is a need for resources on how to think about their property(ies) as coherent systems.

Requests made by individual programs for resources:

- To support more detailed reporting on asthma-related events in schools to minimize the burden of reporting for school nurses/health staff;
- To help educate providers about evidence-based asthma care guidelines, e.g., the National Asthma Education and Prevention Program’s Expert Panel Reports [16];
- To include a clearinghouse of multi-lingual asthma patient education materials;
- To include videos for children on prevention topics, such as home asthma trigger mitigation.

## 4. Discussion

State-level asthma programs currently funded by the CDC serve about two-thirds of the U.S. population [11,17]. Thirty-four program websites were identified and reviewed, and 14 program officials were interviewed. State-level asthma programs provide a wide variety of prevention and management activities. The various interventions for individuals, their homes and families, as well as community-level work in schools and healthcare settings align with expert guidance and the work of asthma programs globally [1,2,3,4,9]. Interventions such as home-visits to mitigate asthma triggers and education on self-management have been shown to have positive returns on investment [18]; these interventions were commonly reported.

Most programs rely on federal agencies and national non-governmental organizations for asthma information. The commonly reported needs were for materials at or below the sixth-grade reading level and videos to share with young patients. These materials should be available in multiple languages; many programs were concerned that materials be culturally appropriate for the ethnic and tribal populations they serve. These needs align with Healthy People 2030 and the expanded definition of health literacy that includes both individual user and health organization roles [19].

To explore the concerns around availability and accessibility of education and outreach materials, a search of the publicly available Asthma Community Network Resource Bank was conducted [20]. (The Asthma Community Network is a collaborative effort of the US EPA, the Robert Wood Johnson Foundation, and the Merck Childhood Asthma Network [21].) The Resource Bank’s advanced search utility was used to document the counts by type, reading level, age group, and language.

The Resource Bank contains a total of 708 items including 133 (19%) that are at or below the sixth-grade reading level (search conducted on 9 July 2024). There are 29 videos in the Resource Bank. Of the 29 videos, 5 (17%) are categorized for children, 6 (21%) for adolescents, and 4 (14%) for children or adolescents. Table 2 summarizes the numbers of asthma education materials available in different languages and at or below a sixth-grade reading level. Items in English (n = 493) or Spanish (n = 175) are the most common. Vietnamese (n = 13), Chinese (n = 8) and Creole (n = 4) round out the top five languages; several languages have only a single item available. On average about 25% of available materials (any language) are at or below the sixth-grade reading level, as recommended by the National Institute of Health for health-related materials [22]. This example highlights the critical need for culturally appropriate accessible materials, particularly as about 8% of the U.S. population speaks English ‘less than very well’ and regionally there is great diversity in languages spoken [23].

## 5. Conclusions and Recommendations

CDC funding for state-level asthma programs is a critical resource for asthma management and prevention. About one-third of the U.S. population is not served by a CDC-funded program. This report focused on state government asthma programs; other organizations, such as local health departments and state chapters of national associations may offer asthma materials or programs.

The work assessed asthma program activities through a website review and in-person interviews. The recommendations reported below were developed from the integrated interview findings. Important limitations of this study include the potential for incomplete information about program goals and activities because websites may not contain full program details or be updated regularly, so the website content may not reflect current work. We attempted to fill information gaps through the interviews. Only one non-CDC-funded program responded to the interview request, so the information needs of these programs are largely unknown. About half of the CDC-funded programs responded to the interview request—this may be due to competing priorities given that many health departments are underfunded and understaffed [24]. CDC-funded programs may not differ greatly due to the structure of grant requirements which mitigates concerns about the small number of interviews.

CEHRT centers are charged with science translation for children’s environmental health; the information needs of state-level asthma programs may be informative for the NIH in designing future CEHRT grant opportunities and for targeting existing CEHRT pilot funding and other programs. Communication and research recommendations included:

- A priority need for improved accessibility of asthma education and outreach materials (readability, language diversity, video format).
- An interest in information to serve potentially vulnerable populations, including young adults and incarcerated individuals.
- A need for outreach materials for the management of asthma triggers tailored to important stakeholders, such as property owners or managers.
- Research needs, such as information on the child to adult transition for asthma patients and on adult asthma more generally.

## Figures and Tables

**Table 1:** Summary of program website content.

Program Goals and Activities	CDC Funded (*n* = 24) No. (%)	Non-CDC Funded ^a^ (*n* = 10) No. (%)
Goals		
Reduce morbidity/mortality	24 (100%)	2 (20%)
Track and reduce disparities/Advance equity	11 (46%)	2 (20%)
Improve quality of life	9 (38%)	2 (20%)
Reduce ED visits and hospitalization	6 (25%)	2 (20%)
Improve self-management and treatment	6 (25%)	5 (50%)
Reduce school absenteeism	1 (4%)	2 (20%)
Improve accessibility and affordability of care	1 (4%)	3 (30%)
Activities		
Provide education and resources	24 (100%)	5 (50%)
In-home outreach/visit	22 (92%)	3 (30%)
Surveillance system	20 (83%)	1 (10%)
Community outreach	16 (66%)	0
Develop policies and guidelines	15 (63%)	2 (20%)
Reduce environmental triggers	10 (42%)	5 (50%)
Improve indoor air quality/reduce tobacco use	10 (42%)	4 (40%)
School or childcare-based outreach	8 (33%)	2 (20%)
Develop collaborations	7 (29%)	5 (50%)
Provider outreach	4 (16%)	1 (10%)
Percent Engagement (Average)	48%	27%

^a^ At least one non-CDC-funded program that is no longer operating still has a website. Abbreviations: ED, emergency department.

**Table 2:** Asthma materials by language in the Asthma Community Network Resource Bank ^a^.

Language	Number of Items	Items at or Below Sixth-Grade Reading Level No. (%)
Arabic	0	0
Chinese	8	2 (25%)
Creole	4	1 (25%)
English	493	120 (24%)
Filipino	0	0
French	1	0
German	0	0
Italian	0	0
Korean	1	0
Native American—Anishinaabe ^b^	1	0
Native American—Lakota ^b^	1	0
Native American—Navaho ^b^	1	0
Russian	0	0
Spanish	175	61 (35%)
Vietnamese	13	2 (15%)
Somali	1	1 (100%)

^a^ Searched on 9 July 2024. ^b^ A public service announcement for radio was translated into these languages.

## Data Availability

The original contributions presented in the study are included in the article/Supplementary Material, further inquiries can be directed to the corresponding author.

## Supplementary Materials

The following supporting information can be downloaded at: https://www.mdpi.com/article/10.3390/ijerph21121670/s1, Table S1: Program URLs, Spreadsheet S1: Website Survey Data, Document S1: Interview Notes.

## Appendix A

Website review questions:

- Name of program
- Start date of program
- Program components
  ○ Mission, Aims and/or Goals
  ○ Program origins, note scientific content or background information
  ○ Note any asthma statistics listed
  ○ What does the program do (how does it achieve its mission)?
  ○ Identify and download any program reports or evaluations

## Appendix B

Interview Discussion Guide:

- Introductions. Describe the BREATHE Center. Describe the project. Review the consent script and remind them that participation is voluntary.
  ○ BREATHE (Bridging Research, Lung Health and the Environment) Center is dedicated to pulmonary health and impacting lung-related health and health disparities through research, community engagement, and advocacy. By focusing on quality research and transparent scientific findings, we strive to engage the community through education efforts. Competitive pilot funding opportunities are available from the center periodically.
  ○ This Project is aimed at learning about state-level asthma programs and how you obtain new information about asthma or asthma programs. We will use the information from this study to develop recommendations about how to communicate information to asthma program staff. We hope this knowledge will improve the BREATHE Center’s communication about asthma and help to prevent asthma morbidity and mortality.
- Review/fill-in gaps in the profile. Any updates? For example,
  ○ What is the source of funding or other sources non-CDC?
  ○ Are you using the EXHALES strategy?
  ○ Is the program monitored/evaluated? If yes, request the most recent report if it can be shared.
- Other questions for program contact
  ○ How do you gather new information on asthma or on your program? What is the best way to get new information?
  ○ If they mention scientific meetings or journal articles, ask them to identify the meeting and/or journal. E.g., American Public Health Association Annual Meeting, Environmental Health Perspectives journal.
  ○ Would you like a copy of the project report emailed to you?
