# Supplementary material for: Information Needs of State-Level Asthma Programs: Recommendations to Increase Accessibility
Source: Int J Environ Res Public Health. 2024 Dec 14;21(12):1670. doi: 10.3390/ijerph21121670 (PMC11675306; doi:10.3390/ijerph21121670)
Supplement: Supplementary file 1 [file ijerph-21-01670-s001.zip › Document_S1.pdf]

Michigan Asthma Program

- **Funding**: The Michigan Asthma Program primarily relies on CDC funding. In the past, it received state funding, but that funding ceased around 2010. Over the years, the program has obtained some other grants but currently depends mostly on CDC funds for its operation.
- **Collaborations**: The program works with multiple partners, including the Asthma Collaborative of Detroit and various school action groups to provide case management and asthma care. Although there was previously a state asthma coalition, it no longer exists. The program has an informal advisory group that provides input for CDC continuation applications.
- **Program Activities**: Despite limited funding, the program is highly valued for its role in coordinating partners and providing technical assistance to asthma care initiatives across the state. These activities often happen without additional funding. Key activities include home asthma case management, connecting partners to resources, and engaging in advocacy for asthma care improvements.
- **Evaluation**: The program employs an external contractor for evaluations, which includes creating a strategic evaluation plan and individual evaluation plans for various initiatives.
- **Challenges**: One of the major challenges the program faces is the limited number of school nurses in Michigan. This presents difficulties in managing asthma-related emergencies in schools, especially with the implementation of a school stock albuterol law. There are concerns about who would assess and administer the medication in emergencies if a nurse isn't available.
- **Educational Needs**: The program identified a need for more educational materials in multiple languages, especially for Michigan’s culturally diverse populations. Additionally, the program seeks an asthma action plan that can integrate with electronic health records (EHRs) to streamline care.
- **Information Sources**: The program gathers asthma-related information through listservs, journal articles, and partnerships. They also have their own listserv, which reaches nearly 6,000 recipients, and they disseminate relevant research and resources through this channel.
- **Scientific Meetings**: Although budget constraints prevent program staff from attending scientific meetings, they still receive valuable digital information from these meetings and distribute it as needed.

Minnesota Asthma Program

- **Program Overview**: The Minnesota Asthma Program focuses on improving the quality of life for individuals with asthma, with a particular emphasis on reducing disparities in emergency department (ED) visits and hospitalizations. The program uses county-level and zip-code-level data to identify and target areas of disparity, particularly in the Twin Cities, where asthma ED visit rates can be six to ten times higher than the state average.
- **Data Usage**: The program utilizes data on ED visits and hospitalizations to identify areas with the highest disparities. They are expecting more granular zip-code-level data statewide to further improve their targeting efforts. They also collect prevalence data and survey data, including the Behavioral Risk Factor Surveillance System (BRFSS) callback survey and the Minnesota Student Survey, to better understand asthma rates and triggers.
- **Policy and Guidelines**: The program works on developing and promoting guidelines for asthma care and focuses on expanding home visits, especially in rural and underserved areas. They have successfully passed legislation that provides coverage for asthma home visits and medical equipment for children under Medicaid, though implementation has been challenging due to coordination issues with various partners.
- **Outreach and Interventions**: Outreach efforts include school-based programs, clinic partnerships, and community outreach in areas with high asthma disparities. The program has provided scholarships for asthma education to nurses and partnered with the American Lung Association to implement guideline-based care in clinics. A recently hired nurse is focused on outreach to schools and clinics, particularly in underserved communities.
- **Challenges**: Implementing the enhanced asthma care legislation has been difficult due to logistical barriers in providing durable medical equipment. Additionally, some clinics face challenges in diagnosing asthma due to a lack of essential equipment, such as spirometers.
- **Evaluation**: The program does not have an external evaluator but conducts internal evaluations. However, the last part-time evaluator was hired away by the CDC, and the program is now seeking a combined epidemiologist and evaluator to fill this gap.
- **Funding**: The program is primarily funded by the CDC cooperative agreement and block grants. They are actively looking for additional funding sources, but asthma is not currently a "hot" topic for funding opportunities.
- **Preferred Information Sources**: The program prefers to receive new asthma-related information through peer-reviewed journal articles rather than webinars or scientific meetings.

Missouri Asthma Program

- **Program Origins**: The Missouri Asthma Program started in 2001 with initial funding from a CDC planning grant, which helped establish a state asthma coalition and a strategic plan. The coalition continued its work until 2008, after which the program shifted its focus to regional and targeted partnerships instead of state-level initiatives.
- **Funding and Partnerships**: Missouri's asthma program is mainly funded by the CDC. In addition to this, the program helps partners such as schools, health centers, and organizations apply for additional grants, as the program itself has limited funds. Foundations such as the Missouri Foundation for Health and the Health Forward Foundation in Kansas City have also supported various asthma-related projects across the state.
- **Focus Areas**: Initially, the program concentrated on pediatric asthma and worked closely with school nurses. Over time, the program expanded its scope to include initiatives like the Extension for Community Healthcare Outcomes (ECHO) program, which focuses on asthma education and management. They have also developed initiatives targeting Federally Qualified Health Centers (FQHCs) and Medicaid recipients.
- **Data Use**: The program has a strong partnership with Medicaid, using claims data to track asthma-related outcomes, such as emergency room visits and medication use. The program produces reports to help clinics improve their asthma care by monitoring their performance.
- **Innovative Projects**: The program is involved in several innovative projects, including the Asthma Risk Panel Report, which assesses asthma management at the clinic level through Medicaid data. Another initiative involves a partnership with NuvōAir to provide inhaler sensors for monitoring medication usage and airflow at home for children on Medicaid.
- **Medicaid Collaboration**: Missouri has made significant strides in asthma care through its collaboration with Medicaid. For instance, the program successfully introduced SMART therapy (single maintenance and reliever therapy) for asthma patients, replacing less effective medications like Singulair.
- **Challenges and Successes**: One of the challenges faced by the program includes the difficulty of obtaining participation in environmental assessments and ensuring coordination between schools and healthcare providers due to regulatory hurdles, such as HIPAA and FERPA. However, the program has seen success in getting Medicaid to fund home environmental assessments and in making pediatric asthma a qualifying condition for health homes.
- **Educational Focus**: The program has created educational materials and is currently focusing on developing short, two-minute videos for outreach targeting patients and families. They continue to explore ways to bridge gaps in healthcare access, such as providing resources for families through community health workers and addressing digital disparities.
- **Relationships**: The success of the program is largely attributed to long-standing relationships with state and local partners, including Medicaid, the University of

Missouri, and other health organizations. These partnerships have allowed the program to sustain its initiatives and integrate asthma care into broader public health efforts.

Pennsylvania Asthma Program

- **Program Overview**: The Pennsylvania Asthma Program is funded through five-year cycles from the CDC, but the program only received four years of funding starting in September 2020 due to a gap in funding. This caused a delay in implementation. The primary focus of the program is to reduce emergency department visits and hospitalizations due to asthma, particularly among children.
- **Funding Sources**: The primary source of funding for the program is the CDC. Additionally, the program receives funds from the Preventative Health and Health Services Block Grant. There are no state funds allocated for the program, and if CDC funding is cut, the position held by the representative would also be eliminated.
- **Program Goals**: The program's goals align with CDC's overarching objectives, which focus on reducing emergency department visits, increasing controlled asthma management, reducing hospitalizations, and decreasing asthma-related deaths. The program also aims to reduce asthma disparities and promote comprehensive asthma services across the state.
- **Activities and Challenges**: The program promotes asthma self-management education and has some home visiting initiatives, along with smoking cessation programs. However, efforts to work with schools have faced challenges due to the COVID-19 pandemic. Policy work, such as the Clean Indoor Air Act and stocking albuterol in schools, is ongoing but has been progressing slowly.
- **Evaluation and Partnerships**: The program's evaluation is conducted externally, with regular updates to assess progress and recommend next steps. Pennsylvania also has an asthma coalition, the Pennsylvania Asthma Partnership, which was reconvened in 2021 and involves around 21 organizations. The coalition is focused on developing strategic plans and moving forward with asthma-related goals.
- **Policy and Advocacy**: The program is involved in policy advocacy, including efforts to close loopholes in Pennsylvania's Clean Indoor Air Act and to promote the stocking of albuterol in schools. The program is also working to ensure that home visiting services are covered by Medicaid and to reduce barriers to accessing asthma medications.
- **Information and Resource Needs**: The program seeks better tracking of trained asthma educators and facilitators, particularly across states, to utilize existing resources. They also expressed the need for more guidance on improving school access for asthma programming and building community engagement. The program would benefit from learning about resources and lessons from other states regarding policy implementation and asthma care.
- **Future Considerations**: The program representative emphasized the importance of addressing the asthma burden in broader contexts, such as housing quality and the

impact of racism, in order to tackle the root causes of health disparities. The program is exploring ways to integrate these broader determinants of health into future asthma program planning.

Texas Asthma Program

- **Program Overview**: The current iteration of the Texas Asthma Program began in September 2019 after a five-year gap due to lack of funding. The program was restarted with a CDC grant, and before the funding gap, a previous version of the program existed until 2015. The program's primary focus is on reducing the asthma burden in Texas, with a particular emphasis on high-risk populations.
- **Funding**: The Texas program relies solely on CDC funding for its asthma-related activities. Other internal resources, such as epidemiologists and evaluators, are shared across different programs through in-kind support, but no additional external funding is available specifically for asthma.
- **Disparities**: The program is committed to addressing health disparities by focusing efforts on populations with the highest asthma burden. These include low-income communities and Black Texans, who experience higher rates of asthma-related hospitalizations. The program also works with schools to reduce disparities through various interventions, including asthma education and providing medications like albuterol.
- **Program Activities**: The program contracts with local health departments in cities like Dallas, San Antonio, and Harris County to conduct asthma education, home visits, and awareness campaigns focused on environmental triggers. School-based activities have included asthma education for nurses and children, with a particular focus on managing asthma to reduce school absenteeism. Virtual outreach and education are also provided due to COVID-19 restrictions. Ongoing efforts include improving provider education through webinars and presentations on updated asthma guidelines.
- **Evaluation**: The program has an internal evaluator who regularly assesses the program's activities and prepares reports for the CDC. However, these evaluations are not typically made public unless they receive specific clearance for broader distribution.
- **Challenges**: Tracking specific asthma-related outcomes, such as school absenteeism, has been difficult due to a lack of comprehensive data. Affordability and access to asthma care are mainly addressed by referring families to insurance options like Medicaid, but there is no direct financial assistance for asthma medication.
- **Collaboration**: The Texas Asthma Program collaborates with various other programs within Texas, such as the tobacco prevention and control program, and facilitates the Texas Asthma Control Collaborative. The program participates in local and regional asthma coalitions and has shared insights with other states, such as New Hampshire and Florida, on program implementation and asthma management.

- **Resource Needs**: The program expressed a need for easy-to-understand educational materials about asthma, written at a sixth-grade reading level, in both English and Spanish. They also desire videos on asthma management. In addition, they seek more resources on business case analysis to help demonstrate the cost-effectiveness of their interventions.
- **Preferred Information Channels**: The program prefers receiving new information through a mix of email lists, journal articles, and national organizations like the American Lung Association. They also attend virtual meetings and webinars, as out-of-state travel is restricted.

Wisconsin Asthma Program

- **Program Overview**: The Wisconsin Asthma Program began in 2001 with CDC funding, following an initial year of partnership building between asthma champions in the state. The primary organizations involved were the Wisconsin Department of Health Services and Children's Health Alliance of Wisconsin. Some program staff joined more recently, in 2021 or 2022.
- **Funding**: The program is primarily funded by the CDC and Medicaid. Recently, the program received a three-year EPA grant for community air monitoring in asthma hotspots in Milwaukee, which started in May 2023. Medicaid funding was also awarded in late 2021, with full program operations beginning in 2022.
- **Program Activities**: The program focuses on in-home asthma education through the Asthma Safe Homes Program, which includes home assessments and remediation services. There is also a strong emphasis on school-based outreach, with educational visits to schools and childcare centers. The program works closely with healthcare providers by offering educational webinars and promoting national asthma guidelines to ensure better clinical care. There is also a strong collaboration with partners like the American Lung Association, the EPA, and various community organizations to support asthma care and prevention efforts.
- **Evaluation**: The program conducts internal evaluations through a part-time evaluator who analyzes the effectiveness of the program's activities. Publicly available evaluation reports are limited, but the program uses data collected through an in-house epidemiologist to guide its activities. This includes tracking asthma prevalence, emergency department visits, and Medicaid data.
- **Environmental Focus**: Environmental triggers for asthma are a key focus for the program. It is involved in air quality monitoring and policy advocacy aimed at reducing exposure to pollutants. Ongoing efforts include anti-idling policies and supporting the enforcement of smart therapy guidelines through Medicaid.
- **Addressing Disparities**: The program uses surveillance data to identify asthma hotspots in the state and prioritize areas where intervention is most needed. It aims to reduce asthma-related health disparities by focusing efforts on the communities most affected.

- **Policy and Advocacy**: The Wisconsin Asthma Coalition is actively involved in advocating for improved asthma care policies and supports public health campaigns related to air quality and smoking prevention. The coalition also advocates for the inclusion of asthma medications, such as smart therapy, under Medicaid coverage.
- **Educational Needs**: The program has expressed a need for patient-facing educational materials, particularly simple, engaging resources such as videos and interactive materials. The goal is to educate both children and families, especially through school channels, about asthma management. School nurses are often overwhelmed, so the program believes that materials that can be easily shared through newsletters or school websites would be helpful. Additionally, the program expressed interest in resources to help asthma patients advocate for themselves during healthcare visits, ensuring they know what to ask their providers.
- **Information Gathering**: The program gathers information from its in-house epidemiologist and relies on partnerships with organizations such as the American Lung Association, the CDC, and the EPA to stay updated on asthma trends. They also use local prevalence data and other emerging trends to guide their efforts.

Puerto Rico Asthma Program

- **Program Overview**: The Puerto Rico Asthma Program began in 2003 and operates as part of the Puerto Rico Department of Health. It is primarily funded by the CDC and focuses on a combination of data-driven strategies, collaborations, and interventions aimed at reducing asthma-related health issues across Puerto Rico. The program's initiatives are guided by the CDC's EXHALE strategy.
- **Funding**: The program is funded by the CDC, and they have also explored collaborations with the EPA, although no additional funding from the EPA has been secured.
- **Collaborations**: The program has established strong partnerships with the Puerto Rico Department of Education to deliver asthma education in schools. This includes initiatives such as the American Lung Association's "Open Airways for Schools" program. The program also collaborates with health insurance providers to gather data on asthma-related emergency department visits and claims. Additionally, healthcare providers, including physicians and nurses, are involved in asthma management education and the proper use of asthma devices.
- **Community Outreach**: One of the flagship community interventions run by the program is the BS project, a home-based asthma service that includes environmental assessments and educational interventions for families affected by asthma. These home visits are designed to identify and mitigate environmental triggers that exacerbate asthma. The program also conducts public education campaigns using media and direct community engagement to raise awareness about asthma management and environmental health.
- **Program Evaluation**: The program conducts internal evaluations to track the effectiveness of its initiatives. Evaluations focus on monitoring asthma-related

emergency visits, hospitalizations, and the overall quality of asthma management interventions provided. Evaluation reports are shared with the CDC, though it is unclear whether they are made available to the public.

- **Challenges**: A major challenge for the program has been ensuring that healthcare providers, particularly in schools and hospitals, adhere to national asthma management guidelines. Additionally, the program has encountered obstacles in accessing more sustainable funding sources outside of the CDC, which limits the expansion of certain interventions.
- **Resource Needs**: The program expressed a desire for more tracking and training resources for asthma educators and facilitators, particularly to identify where such trained professionals are located across the island. There is also a need for educational materials in both English and Spanish, especially for patient and community-facing interventions. Additionally, the program is interested in receiving more guidance on building relationships with schools and community organizations to further asthma education and management in underserved areas.
- **Future Directions**: The program aims to improve asthma surveillance across Puerto Rico to better understand disparities in asthma outcomes. This includes investigating broader determinants of health, such as housing quality and environmental exposures, that may contribute to asthma issues. The program also plans to focus on addressing health inequities by targeting asthma interventions to the most affected communities, particularly those that are economically disadvantaged.

Maine Asthma Program

- **Program Overview**: The Maine Asthma Program follows the CDC's EXHALE strategy and is primarily funded by the CDC. In addition to CDC funding, Maine also receives some public health funding through the state's tobacco settlement, which supports the program. The program's efforts focus on reducing asthma-related hospitalizations and emergency department visits across the state.
- **Data and Evaluation**: The program works with the Maine Tracking Network to gather and analyze asthma-related data, such as emergency department visits and hospitalizations. Due to delays in the availability of data, the program is usually one to two years behind on certain data points. While the program conducts external evaluations, there is no formal internal evaluation process in place. However, the team consistently evaluates the effectiveness of their interventions through informal feedback and performance assessments.
- **Funding and Collaborations**: Although the program does not receive much external funding beyond the CDC, it partners with other state agencies, such as the tobacco program and the childhood lead poisoning prevention program. The Maine Asthma Program also collaborates with organizations like the Maine Indoor Air Quality Council and local housing agencies to support joint efforts related to home repairs and improving air quality.

- **Program Challenges**: One of the major challenges in Maine is educating property owners and managers about how to maintain healthy homes, particularly in low-income and rural areas. Many property owners lack the necessary resources or knowledge to properly maintain their properties, which can exacerbate asthma problems for residents. The program is actively seeking more proactive ways to address poor housing conditions before asthma becomes a more serious issue.
- **Addressing Disparities**: The program places a strong emphasis on reducing asthma disparities by targeting high-risk populations, including recent immigrants and rural residents. To expand asthma self-management education, the program aims to utilize community health workers and community paramedic programs to reach more of these underserved populations. However, rural access to asthma care remains a significant barrier in Maine, making it difficult to provide care to all affected individuals.
- **Information Gathering**: The program gathers information from various sources, including updates from the CDC, the American Allergy and Asthma Foundation, and a regional group of New England states focused on asthma care. Additionally, the program uses alerts from scientific journals and shares resources through the state's asthma coalition to keep up with new developments in asthma care.
- **Resource Needs**: The program has expressed a need for more educational materials geared toward property owners, landlords, and homeowners, specifically on how to maintain healthy homes with a focus on environmental health and asthma triggers. These resources would be particularly helpful in addressing asthma disparities caused by poor housing conditions.

Georgia Asthma Program

- **Partnerships and Collaborations**: The Georgia Asthma Program collaborates with a variety of statewide partners, including health systems, school systems, and environmental organizations. The Georgia Asthma Advisory Board, which was established in 2015, oversees the program's strategic plan. Additionally, the program operates various workgroups focused on specific areas, such as environmental interventions, school and childcare settings, and health systems.
- **Program Evaluation**: The program conducts internal evaluations through a full-time evaluator who is part of the staff. Evaluations are done on a project basis, not annually, and follow a strategic evaluation plan. The evaluation reports are not currently available for public sharing, as they must go through an approval process before being released.
- **Resource Needs**: The Georgia Asthma Program identified a need for more educational materials in multiple languages, particularly for the state's diverse populations, including Spanish speakers in rural areas and other language groups in Metro Atlanta. The program also expressed interest in using different formats for educational materials, such as videos, mobile apps, and websites, to better reach their target audiences.

- **Information Sources**: The program primarily relies on guidance from the CDC for evidence-based practices and recommendations. Program staff stay informed through CDC updates, as well as studies on specific topics such as the relationship between gas stoves and asthma. Although the program relies heavily on CDC resources, it also turns to journal articles when specific topics, like the relationship between asthma and COVID-19, arise.
- **Resource Needs**: In addition to the need for multilingual resources, the program is interested in expanding its use of digital platforms for education, including apps and websites, to better engage and inform asthma patients and caregivers.

New Hampshire Asthma Program

- **Program Overview**: The New Hampshire Asthma Program has been operating for over 20 years and is fully funded by the CDC. The program follows the CDC's EXHALE strategy but faces limitations due to the lack of funding beyond CDC support, making it difficult to implement a wide range of activities.
- **Evaluation**: The program uses external evaluators for specific projects rather than conducting annual evaluations. The evaluations are not made publicly available but are shared with the CDC as part of the cooperative agreement's reporting requirements. These reports are submitted annually to the CDC as part of the five-year cooperative agreement.
- **Collaborations and Partnerships**: The program collaborates with several organizations, including Mobile Integrated Health (MIH) programs for home visits, the American Lung Association, and the New England Chapter of the Asthma and Allergy Foundation. Additionally, they are starting new collaborations with camps like "Super Crossings," which focus on families of children with asthma.
- **Program Activities**: New Hampshire previously had home visiting programs through local health departments, but these were discontinued due to sustainability issues. The program is now piloting a home visit model using mobile integrated healthcare. The program also engages in provider-based outreach, such as training school nurses and collaborating with local healthcare systems. However, staffing shortages and the ongoing impact of COVID-19 have made it challenging to expand these efforts. Educational efforts include plans for teacher training on managing asthma in classrooms and promoting the "Open Airways" program for students.
- **Barriers to Success**: One of the significant challenges is finding healthcare partners willing to take on the referral process for home visits. Healthcare providers face numerous barriers, including staff time and resource constraints, making it difficult to integrate asthma services into their systems. Additionally, the administrative structure in New Hampshire limits the number of grants that can be applied for due to capacity restrictions on managing these funds at the state level.
- **Information Gathering**: The program stays informed through webinars, newsletters, and publications from organizations like the American Lung Association and the

Asthma and Allergy Network. They also refer to journals like *Allergy and Immunology* for scientific updates and to stay current with asthma care developments.
- **Resource Needs**: The program expressed a need for updated educational materials geared toward families and children, particularly materials written at an accessible reading level. Much of the available material is either outdated or written at too high a literacy level for the target audience. Additionally, the program faces restrictions on providing durable medical equipment, such as inhalers or spacers, which limits their ability to assist asthma patients directly.

Montana Asthma Program

- **Program Overview**: The Montana Asthma Program started in 2007 with state funding from the Master Settlement Agreement dollars (tobacco settlement) and received CDC funding in 2009 through a cooperative agreement. The program has expanded over time, adding more initiatives as additional funding became available.
- **Funding**: The program is primarily funded by the CDC and continues to receive some funds from the Master Settlement Agreement. Similar to other asthma programs, the Montana program faces limitations in funding, restricting its ability to carry out all desired activities. The program has expressed interest in finding ways for payers, such as insurers, to cover services like asthma home visits in order to expand its reach.
- **Program Evaluation**: The Montana Asthma Program follows a strategic evaluation plan that aligns with the CDC's five-year grant cycle. They conduct two to three evaluations annually, focusing on process evaluation. Evaluations are carried out by internal evaluators, and the reports are either published on the program's website or shared with their partners.
- **Collaborations and Partnerships**: The program operates the Montana Asthma Advisory Group, which consists of healthcare providers, school nurses, and public health professionals. This group provides guidance and feedback on the program's direction and initiatives. The advisory group helps shape the program's strategies and may assist with specific projects, though the level of participation varies.
- **Information Sources**: The program stays informed through journal subscriptions, including publications like the *Journal of Asthma* and *Chest*. They also attend national conferences, such as those hosted by the Association of Asthma Educators and the Council for State and Territorial Epidemiologists. Additionally, they participate in forums like the "Asthma Talk" listserv, which allows states to share information about asthma programs and best practices.
- **Resource Needs**: The Montana Asthma Program expressed a need for more educational materials in multiple languages to better serve their diverse population. The program is also interested in creating social media resources that can be easily adapted for various audiences, making asthma education more accessible. Additionally, the program seeks information on how to secure long-term sustainability for asthma programs, including strategies for getting services like asthma home visits reimbursed.

Connecticut Asthma Program

- **Program Overview**: The Connecticut Asthma Program began in 2000 and was first funded by the CDC's National Asthma Control Program (NACP) in 2002. The program has a long history of providing asthma education and management services to the state's residents. Early programs included "Easy Breathing" and "Putting on Airs," both of which focused on home-based asthma education and reducing environmental asthma triggers.
- **Funding**: In addition to CDC funding, the Connecticut Asthma Program receives funds from the Preventative Health and Health Services (PHHS) block grants. However, the program has faced ongoing challenges related to staffing and funding. Due to limited state support, the program has had to rely more heavily on federal funding to cover salaries, which in turn reduces the funds available for actual services and program activities.
- **Program Flexibility**: As part of the CDC's cooperative agreement, the program must meet certain objectives and guidelines, but there is some flexibility in how the program is structured and implemented. For instance, the program had to adapt significantly during the COVID-19 pandemic, moving away from in-person home visits to virtual visits. However, this change impacted the evidence base of the intervention, as virtual visits do not offer the same level of effectiveness as in-person visits.
- **Program Evaluation**: The program has used an external evaluator since 2014. The evaluations focus on the program's impact, although the planned evaluation of their home visiting program was put on hold due to the pandemic. The program is currently conducting an economic evaluation based on pre-COVID data to assess the cost-effectiveness of its interventions.
- **Major Changes Over Time**: Since 2014, the program has benefited from the CDC's EXHALE strategy, which provides a structured approach to managing asthma-related activities. However, the program has experienced turnover in public health personnel, which has brought new perspectives but also posed challenges in maintaining continuity within the program.
- **Information Gathering**: The program stays informed through webinars, journals, and partnerships with organizations like the American Lung Association and the Allergy and Asthma Network. Additionally, the program collaborates with groups working on indoor air quality and children's health to address environmental factors impacting asthma.
- **Resource Needs**: The program highlighted a need for more resources related to air quality and housing, as these areas directly impact asthma but are outside the program's direct control. They are also interested in finding ways to integrate clinical data with public health surveillance data to better understand healthcare providers' adherence to asthma guidelines.

- **Challenges**: The program faces significant challenges in enforcing asthma guidelines among healthcare providers due to a lack of regulatory power. Additionally, there is a shortage of pulmonologists in the state, which puts more pressure on primary care providers to manage asthma cases.
- **Future Directions**: Looking ahead, the Connecticut Asthma Program sees potential in integrating artificial intelligence (AI) into healthcare settings to help providers adhere to asthma management guidelines. The program is also focusing on rebuilding trust with communities, particularly after the COVID-19 pandemic, which strained relationships and made it more difficult to engage families in asthma programs.

Florida Asthma Program

- **Program Overview**: The Florida Asthma Program started in 2009 as part of the CDC's competitive grant process. Since then, the program has undergone three five-year funding cycles, with the current cycle running from 2019 to 2024. The program aims to reduce asthma-related hospitalizations and emergency department visits across the state.
- **Program Funding**: The Florida Asthma Program is primarily funded by the CDC. Additional funding comes from Children's Medical Services, but this is minimal compared to the CDC funds. These supplemental funds are typically used to purchase educational and home-visiting supplies, such as hypoallergenic mattress covers and cleaning supplies, to help manage asthma triggers at home.
- **Program Activities**: The program follows the CDC's EXHALE strategy and adheres to the Expert Panel Report 3 guidelines, which overlap in terms of asthma care management. The Florida Asthma Program runs several initiatives, including the Florida Asthma Home Visiting Program and "Asthma Friendly" initiatives for schools, childcare centers, hospitals, and healthcare providers. These activities focus on reducing emergency department visits, hospitalizations, and improving overall asthma control. Assessments such as the Asthma Control Test (ACT) are commonly used to measure the effectiveness of asthma interventions.
- **Program Evaluation**: The program is evaluated externally by Florida State University (FSU). The evaluator has been working with the program for around 12 years, providing ongoing assessments of the program's activities. Evaluation data is submitted yearly to the CDC, focusing on performance measures like the reduction of emergency visits and hospitalizations. Evaluation reports are not always publicly available and would need approval for sharing.
- **Collaborations**: The program works closely with the Florida Asthma Coalition, which includes professionals from various fields, including doctors, pharmacists, respiratory therapists, and public health workers. These professionals promote asthma education and care across the state. Additionally, the program collaborates with other asthma coalitions, such as the Tampa Bay Asthma Coalition, and occasionally with coalitions from other states, such as Georgia and Louisiana.

- **Resource Needs**: The program expressed a need for more resources related to adult asthma management, as there is confusion among patients regarding the distinction between asthma and other chronic lung conditions like COPD and emphysema. Another area of interest is research on the transition from pediatric to adult asthma care, especially for young adults who are moving away from home and becoming responsible for managing their own asthma.
- **Challenges**: One of the challenges faced by the program is that asthma medications used for multiple lung conditions, like COPD, can create confusion, particularly among adult patients. Additionally, the program highlighted the difficulties faced by incarcerated populations with asthma, especially in managing their condition once they are released from prison.
- **Information Gathering**: The program gathers information from a variety of sources, including newsletters from the National Institutes of Health (NIH), the CDC, and the EPA. They also rely on data from their own home-visiting program to assess the effectiveness of their interventions. Preferred sources of information include both scientific journals and asthma-related conferences, such as those hosted by the Allergy and Asthma Network and the American Academy of Allergy, Asthma & Immunology.

Louisiana Asthma Program

Background: Louisiana is a rural state and most of the areas with poor respiratory outcomes - especially increased rates of asthma prevalence is in more rural areas. There are other components of the state that are further away and we couldn't really get to - rely on virtual home visits.

- **Virtual home visits:** primary mode of education for participants. They do one hour meeting either a physician - training in asthma education or with respiratory therapists who have a lot more experience and are working for 20 years with childhood asthma. It is very cost effective. It would take four hours to drive to some of the people versus just being able to talk to them automatically over the phone.
- The downside to that is not everyone has access to like phones or access to Internet. So that does limit our availability to reach everybody.

- **Education**: They do one hour long first visit going over both asthma medications and environmental triggers and getting a bunch of other background demographic data from the people that are enrolled in the program. And then they meet with them two more times over the phone, ask them how they're doing, ask them if they have any questions and see how their asthma is improving or getting worse and offering any advice available.

- **Funding:** In a period of not a lot of funding - only existing because they are part of the Louisiana Department of Health. Looking to get more funding in July.

Enrolling people who were previously reached through outreach events in the past. Trying to expand outreach in other ways. Aren't getting as many new people for the last couple of months due to lack of resources. our funding in July will be from the Louisiana Department of Health – giving some amount of money to sustain a very small team to keep it running in perpetuity. So at that time, they'll have enough money to fund a asthma educator full time to help continue to reach patients.
Also always like looking for grants. EPA government to government grant that got released, which offers a million dollars over three years. And so that's what they're working on.
Right now, they're running just through volunteer services. It is not that bad, but no one's getting paid, so it's hard to expand the program.

- **Evaluations/Monitoring**: Don't do any formal evaluation process, but do annual executive summaries just to make sure that they're not falling behind and not missing any opportunities.

- **Collaborations**: Our Lady of the Lake Hospital - used to do all the asthma education for everyone and we paid them an amount of money for their respiratory therapist.
  Now they're doing in kind services, which is the asthma education, virtual home visit or in person visit with patients who are part of their patients.

  Louisiana Center for Health Equity, that's another organization that they're tied with. They're a community-based organization - nonprofit.They don't give any funding, but whenever they have community outreach events in Baton Rouge, hand out flyers and try to get people excited about the program

- **Changes:** The biggest change is when it first started, it was right before the COVID-19 pandemic. So they actually ended up getting a bunch of enrollees through our contact tracing. Contact tracers were instructed to ask a question like, do you have asthma? And if someone said yes, then they were automatically enrolled into the program. Had a lot of enrollment early on from people who probably didn't have asthma as badly as they needed to actually use the services. And so they had a lot of people who just kind of never followed through.

  But then other health systems that were interested - Shreveport was really excited and they enrolled. - clinic heard and started enrolling patients. So got a lot of people enrolled that way.

  They had enough funding to provide this really interesting pilot program for like only six families that involved an air purifier, HEPA filter, vacuum and air monitoring. - interesting because it's like indoor air monitoring for homes.

- **Information Resources:** Curriculum was made from the Green and Healthy Homes Initiative - the problem with some of our information, is that it's at a really high reading level - it's very difficult to understand. Create more helpful information.
  Lady of the Lake Children's Hospital made this amazing little book for that has information about how to manage asthma. And I think it is written in like fairly straightforward and easy way. They would always love more materials. Right now it is a bunch of random pamphlets, but they would accept more information, especially if it's already been fact checked by someone else who knows what they're doing. We want things to look really pretty.
- **Information Needs**: Lacking right now is an explanation about what asthma is.

  They would love to have a very simple, instructive information about what asthma is, why it's important - in a simple way so the average reading level in Louisiana is like a fifth or sixth grade level.
  From all the research that is being conducted, the most recent evidence-based information. When you show evidence, it's taken more seriously from a reliable source.

  If we say that these are the numbers and this is how this can help you, that's what attracts a lot of people.

  There is a deficit kind of in the pathophysiology of asthma. Most of the information is more about environmental triggers. No strong information about asthma medications.

  There's not a lot of video stuff. It would be so easy to send people YouTube links. That would be a lot easier than having them read something. So I guess that would be a thing to work on potentially.
