# Supplementary material for: Information Needs of State-Level Asthma Programs: Recommendations to Increase Accessibility
Source: Int J Environ Res Public Health. 2024 Dec 14;21(12):1670. doi: 10.3390/ijerph21121670 (PMC11675306; doi:10.3390/ijerph21121670)
Supplement: Supplementary file 1 [file ijerph-21-01670-s001.zip › Table S1.pdf]

Table S1. Program URLs

State	Current CDC Funding Y/N	Program URL
California	Y	https://www.cdph.ca.gov/Programs/CCDPHP/DEODC/EHIB/CPE/Pages/CaliforniaBreathing.aspx
Connecticut	Y	https://portal.ct.gov/DPH/Health-Education-Management--Surveillance/Asthma/Asthma-Program
Florida	Y	https://www.floridahealth.gov/diseases-and-conditions/asthma/index.html
Georgia	Y	http://dph.georgia.gov/asthma-surveillance
Illinois	Y	https://dph.illinois.gov/topics-services/diseases-and-conditions/asthma/il-asthma-partnership.html
Indiana	Y	https://www.in.gov/health/cdpc/respiratory-health/asthma/
Kentucky	Y	https://www.chfs.ky.gov/agencies/dph/dpqi/cdpb/Pages/asthma.aspx
Maine	Y	https://www.maine.gov/dhhs/mecdc/population-health/mat/
Massachussets	Y	https://www.mass.gov/asthma-prevention-and-control?_gl=1*t384i0*_ga*MTkxMjk5NDQ3Mi4xNzA3NzY2Njcx*_ga_MCLPEGW7WM*MTcwNzc2NjY3OC4xLjAuMTcwNzc2NjY4NC4wLjAuMA..
Michigan	Y	https://getasthmahelp.org/about-aim.aspx
Minnesota	Y	https://www.health.state.mn.us/diseases/asthma/index.html
Missouri	Y	https://health.mo.gov/living/healthcondiseases/chronic/asthma/
Montana	Y	https://dphhs.mt.gov/publichealth/asthma/index
New Hampshire	Y	https://www.dhhs.nh.gov/programs-services/disease-prevention/asthma
New Mexico	Y	https://www.nmhealth.org/publication/view/plan/6655/
New York	Y	https://www.health.ny.gov/diseases/asthma/ny_action.htm
Ohio	Y	https://odh.ohio.gov/know-our-programs/asthma-program/asthma-program
Pennsylvania	Y	https://www.health.pa.gov/topics/programs/Asthma/Pages/Asthma.aspx
Puerto Rico	Y	https://www.cdc.gov/asthma/contacts/asthma-grantee-profile-pr.html
Rhode Island	Y	https://health.ri.gov/programs/detail.php?pgm_id=5
Texas	Y	https://www.cdc.gov/asthma/stateprofiles/Asthma_in_TX.pdf
Utah	Y	**https://health.utah.gov/asthma/about/index.php**
Vermont	Y	https://www.healthvermont.gov/wellness/asthma-lung-disease
Wisconsin	Y	https://www.dhs.wisconsin.gov/asthma/index.htm
Arizona	N	https://www.azdhs.gov/documents/prevention/tobacco-chronic-disease/tobacco-free-az/reports/asthma-control.pdf
Hawaii	N	https://livinghealthy.hawaii.gov/control-asthma-hawaii/ https://health.hawaii.gov/asthma/
Louisiana	N	https://ldh.la.gov/page/BREATHE
Maryland	N	https://health.maryland.gov/phpa/OEHFP/EH/pages/asthma.aspx
Mississippi	N	https://msdh.ms.gov/page/43,0,235.html
New Jersey	N	https://www.nj.gov/health/fhs/chronic/asthma/
North Carolina	N	https://asthma.dph.ncdhhs.gov/
Oregon	N	https://www.oregon.gov/oha/ph/diseasesconditions/chronicdisease/asthma/pages/index.aspx https://www.oregon.gov/oha/PH/DISEASESCONDITIONS/CHRONICDISEASE/ASTHMA/Pages/plan.aspx
Washington	N	https://doh.wa.gov/public-health-healthcare-providers/public-health-system-resources-and-services/local-health-resources-and-tools/local-asthma-coalitions/washington-asthma-initiative
West Virginia	N	https://www.asthmacommunitynetwork.org/node/1034
